# Supplementary figures and images for: Genome-wide identification and characterization of the sucrose invertase gene family in Hemerocallis citrina
Source: PeerJ. 2024 Aug 29;12:e17999. doi: 10.7717/peerj.17999 (PMC11366234; doi:10.7717/peerj.17999)

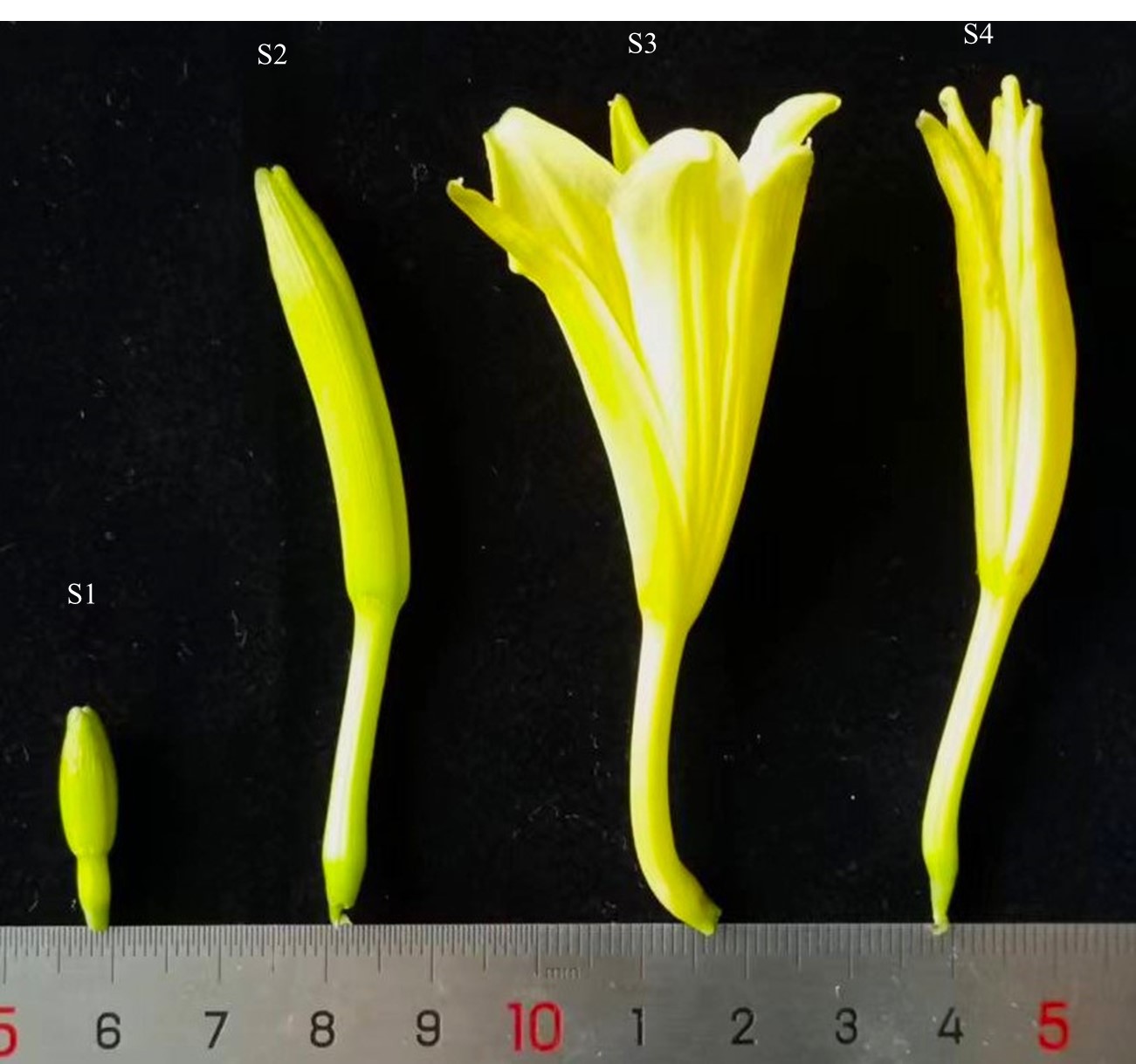

Supplement: Supplemental Information 1 [file peerj-12-17999-s001.jpg]

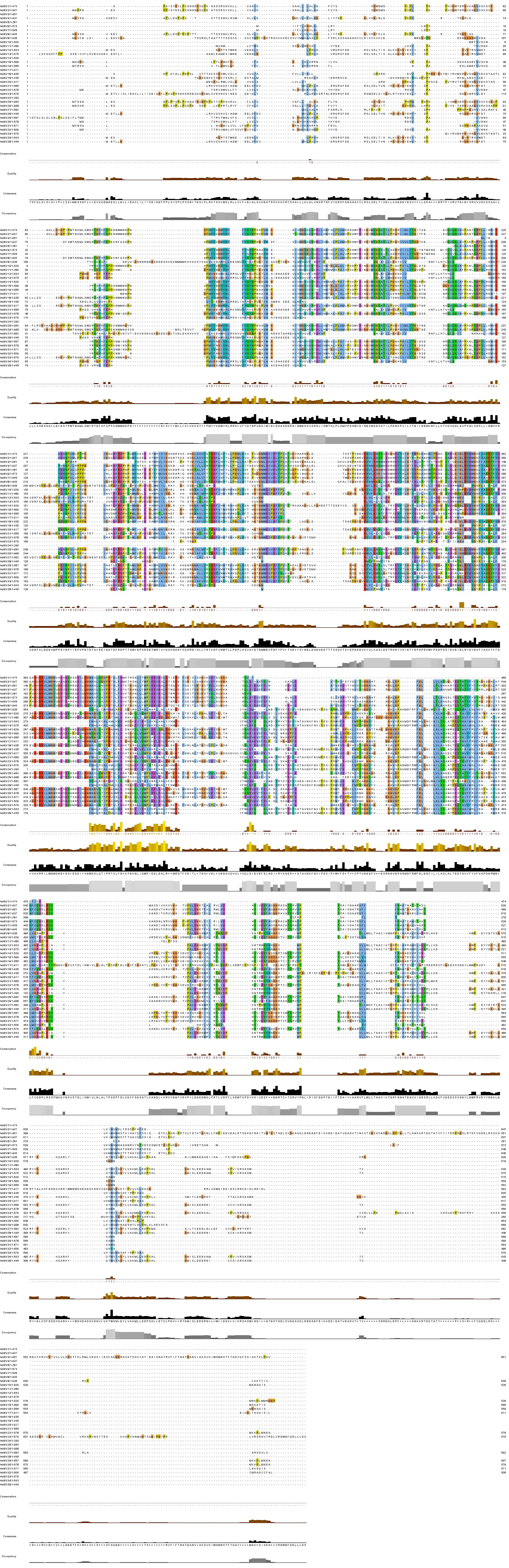

Supplement: Supplemental Information 2 [file peerj-12-17999-s002.png]

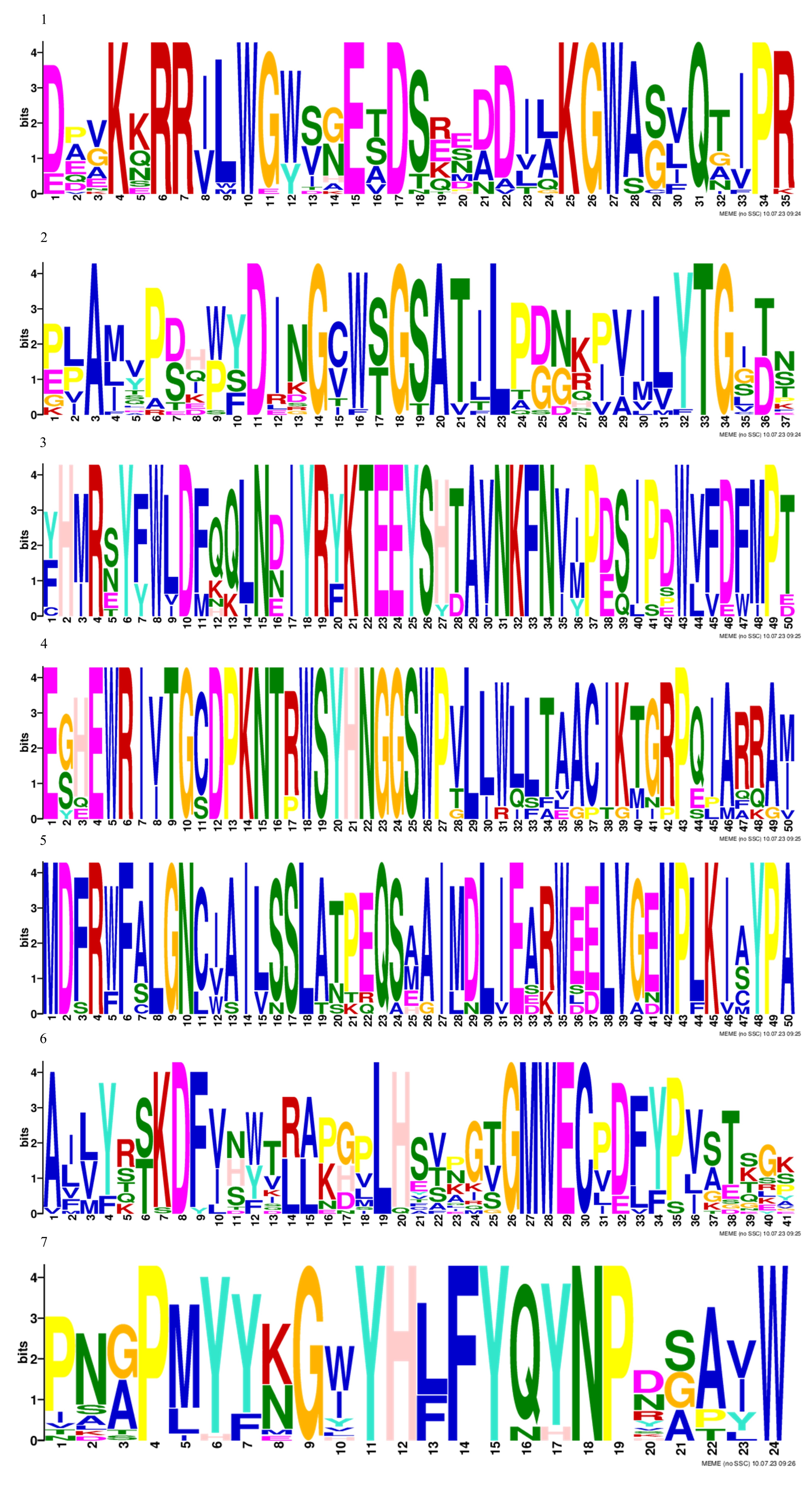

Supplement: Supplemental Information 3 — The motif code is provided above the y axis. [file peerj-12-17999-s003.jpg]

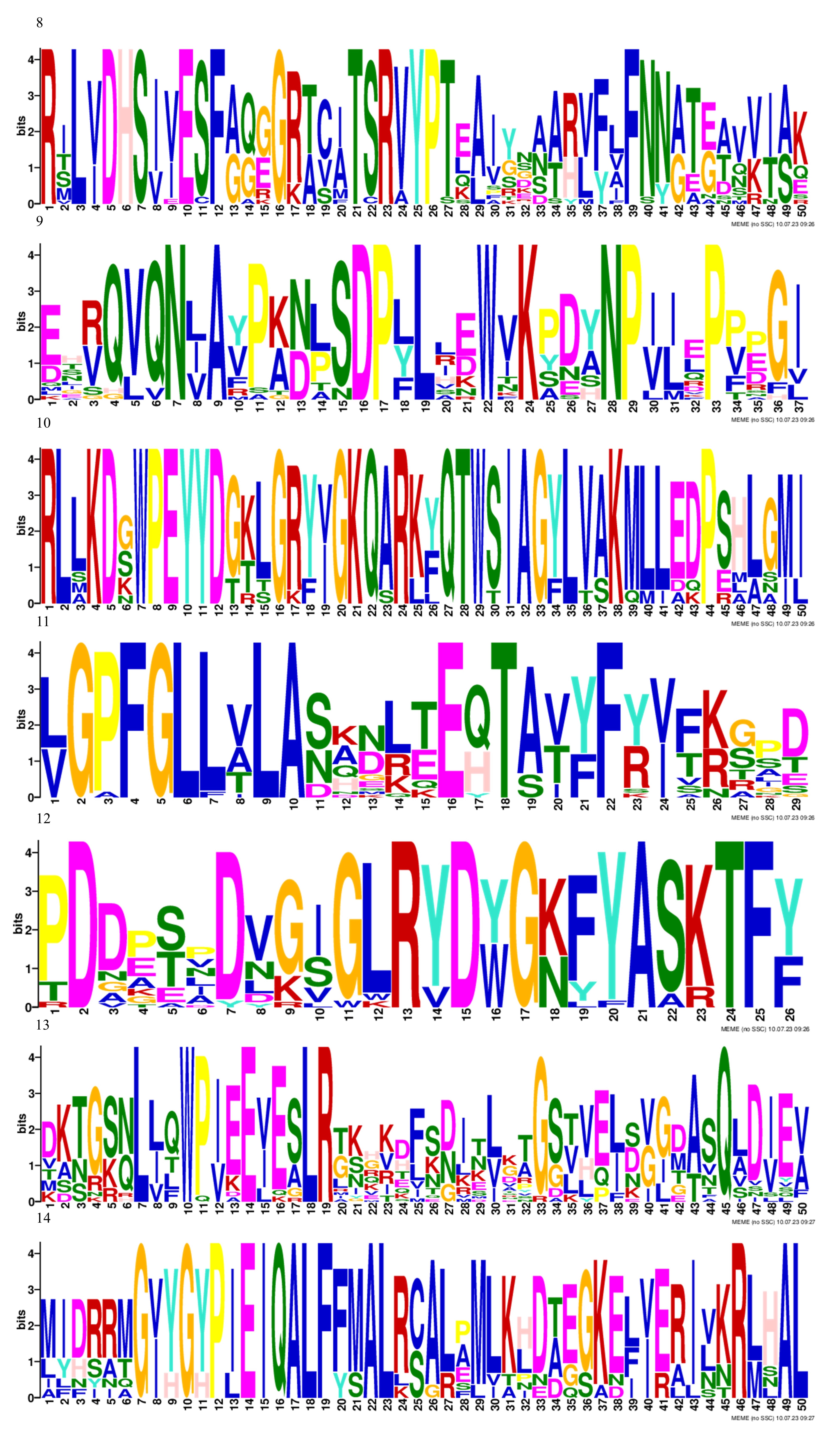

Supplement: Supplemental Information 4 — The motif code is provided above the y axis. [file peerj-12-17999-s004.jpg]

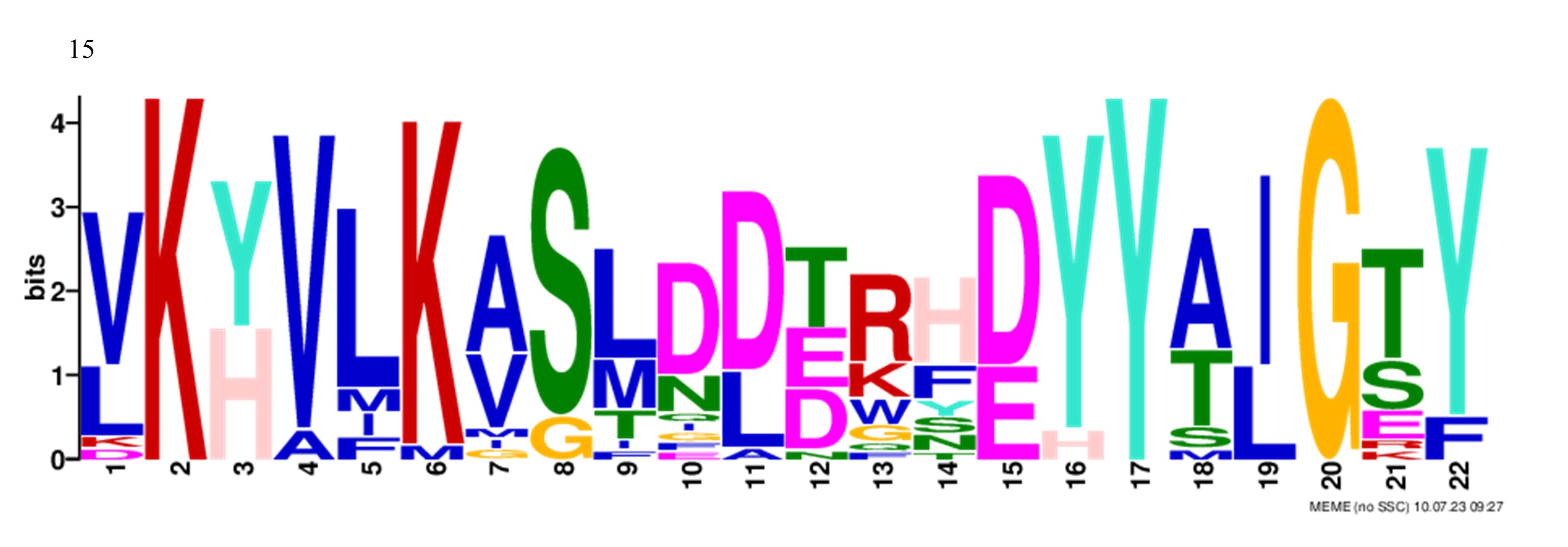

Supplement: Supplemental Information 5 — The motif code is provided above the y axis. [file peerj-12-17999-s005.jpg]

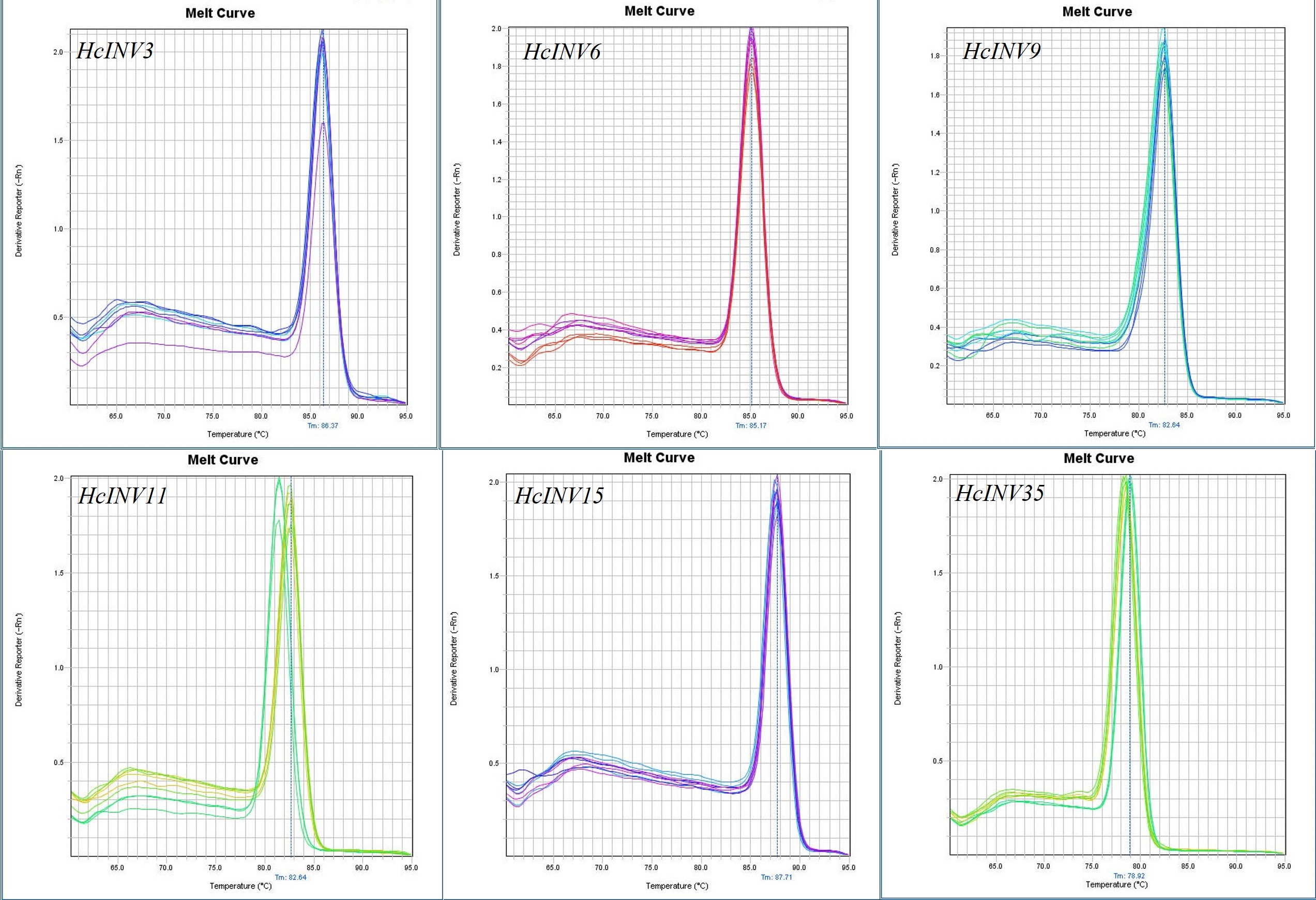

Supplement: Supplemental Information 14 [file peerj-12-17999-s014.jpg]
