# Supplementary material for: Genome-wide identification and characterization of the sucrose invertase gene family in Hemerocallis citrina
Source: PeerJ. 2024 Aug 29;12:e17999. doi: 10.7717/peerj.17999 (PMC11366234; doi:10.7717/peerj.17999)
Supplement: Supplemental Information 6 [file peerj-12-17999-s006.docx]

Supplementary Table 1 Sequences of primers used in this study.

| Gene | Forward primer (5’→3’) | Reverse primer (5’→3’) |
| --- | --- | --- |
| *HcINV3* | GAACGATGAGAGGCACGACT | TGTCATCCTCCCGGCTATCA |
| *HcINV6* | AGTGGGCATCAACGACGAAT | CCTGAGTCCAATGCCAAGGT |
| *HcINV9* | CCAGGTCAAGGTCTGATGCC | TCAACTGGTGCTACACGACC |
| *HcINV11* | GGTTGACAATGGATCGGGGT | ACCCTTGGCAACATCGTCAT |
| *HcINV15* | CGTGAGTGGATCAAACCCGA | CCCGATCCCAAGTCACGAAA |
| *HcINV34* | AGGATTGTCACTGGTTGCGA | CAGGCCAGCCATCTTTCAGT |
| *HcGAPDH* | CTTGAGGCAGTCCCCAACAT | CAAGCAAAACCTGAGGCGAC |
